# Judiciary views on criminal behaviour and intention of offenders with high-functioning autism

**DOI:** 10.1108/JIDOB-02-2014-0002

**Published:** 2013

**Authors:** Colleen M. Berryessa

**Affiliations:** Program Manager, based at Center for Biomedical Ethics, Stanford University, Stanford, California, USA

**Keywords:** Criminal behaviour, Asperger’s syndrome, hfASDs, High functioning autistic spectrum disorders, California superior court judges, Judiciary views

## Abstract

**Purpose:**

The purpose of this paper is to explore how judges perceive High Functioning Autistic Spectrum Disorders (hfASDs) and the disorders’ effects on an offender’s ability to formulate criminal intent and control behaviour.

**Design/methodology/approach:**

Semi-structured interviews on topics related to offenders with hfASDs were conducted with 21 California Superior Court Judges. A coding scheme was developed and an iterative qualitative coding process was used for analysis.

**Findings:**

Analysis yielded three major themes on how an hfASD diagnosis affects an offender’s ability to regulate actions and criminal behaviour. Interviewed judges reported beliefs that hfASD offenders view the world in a different way and that much of their behaviour is not under their direct control. Judges reported these perceptions likely affect how they criminally process and make legal decisions regarding offenders with hfASDs.

**Research limitations/implications:**

The sample size was small and therefore no statistical significance can be drawn from results; findings cannot be applied to perceptions or experiences of the entire California Superior Court Judge population.

**Originality/value:**

Past academic research reports that individuals with hfASDs that offend often do so because of specific symptoms associated with the disorder. This presents a complex dilemma for the criminal justice system regarding how best to understand the disorder and process these offenders. This study and its findings aim to shed light on issues judges encounter in determining these offenders’ responsibility and sentencing, in what ways this information might be integrated into judicial decision making, and areas where future research is needed.

## Introduction

Although there has been academic disagreement about the relationship between Autistic Spectrum Disorders (ASDs) and criminality (for a comprehensive review of this research, see Browning and Caulfield, 2011), most individuals diagnosed with High Functioning Autistic Spectrum Disorders (hfASDs)[1] are law-abiding citizens who rarely commit criminal acts (Ghaziuddin *et al*., 1991; Murrie *et al*., 2002; Woodbury-Smith *et al*., 2006; Browning and Caulfield, 2011). Yet, individuals with ASDs are seven more times likely to encounter the criminal justice system than those without the disorders (Debbaudt, 2004). Although each offender diagnosed with an ASDs is different and should be processed and assessed individually by the court, the majority of individuals with hfASDs who exhibit criminal behaviour are thought to do so as a presentation of or in association with the symptoms of their disorders, specifically related to poor impulse and motor control, narrow fixation on specific interests, theory of mind deficits, and a lack of understanding of social cues, personal space, and the effects of one’s behaviour on others (Murrie *et al*., 2002; Barry-Walsh and Mullen, 2004; Howlin, 2004; Haskins and Silva, 2006; Attwood, 2006; Kristiansson and Sorman, 2008; Browning and Caulfield, 2011). Often, crimes committed by these individuals seem bizarre or unusual in nature and are best understood or explained by looking at the symptomatic features of their disorders (Haskins and Silva, 2006). Diagnosed offenders may be unaware of the harm they cause and not understand why they are facing criminal charges. This calls into question the existence of criminal action and criminal intent in offenders with hfASDs and, correspondingly, offenders’ legal responsibility and the legal consequences they ought to face (Freckelton, 2011).

The possibility that criminal actions may arise from features of the disorders presents a complex set of issues for criminal justice actors – such as judges, juries, attorneys, prison and parole officers, law enforcement, and clinicians or others involved in forensic mental health services – who may need to separate the criminal act and intention of an offender from the symptoms of his disorder or associated personal characteristics in order to properly assess or criminally process an offender and his actions. Additionally, the introduction of research highlighting genetic causes of ASDs (Geschwind, 2011) to legal evaluation could further complicate these issues of criminal action, intent, legal responsibility, and consequences, as it has with other disorders such as schizophrenia (Jones, 2002), addiction (Morse, 2006), and psychopathy (Aspinwall *et al*., 2012). It has been reported that the frequency of cases in which information on ASDs, including hfASDs, and their relevance to criminality is being used in criminal trials is increasing (Freckelton, 2013). Yet, to my knowledge, there is no research on how judges perceive hfASDs, the characteristics of offenders with hfASDs and their relationship to criminal behaviour, intention to commit criminal acts, and culpability. Further, there is very little research on how different mental health factors generally affect judicial perceptions and decisions (Andrews *et al*., 1987; Hoge, 1999; Jones and Cauffman, 2008). Thus, to contribute to this lacking research area as the incidence of criminal cases involving ASDs and hfASDs continues to grow, this paper seeks to add to understandings of judicial perceptions of mental health, focusing on the criminal actions and intentions of offenders with hfASDs, and how these perceptions might be integrated into judicial decision-making surrounding diagnosed offenders.

In general, academic reporting on judicial perceptions of offenders with mental health disorders has discussed how judges’ preexisting opinions or experiences, both positive and negative, of those with mental disorders often affects their interpretation of legal information and decisions in cases involving offenders with mental health disorders or disabilities (Perlin and Gould, 1995; Wrightsman, 1999; Parry, 2005; Jones and Cauffman, 2008). Other research has found that the judiciary has often treated mentally disordered offenders rather leniently, suggesting that mental health factors mitigate judicial perceptions of responsibility, dangerousness, and behavioural control (Hochstedler, 1987). Judges have also been shown to apply more moderate sentences in cases in which they believed an offender’s behaviour had been affected by external factors, such as mental health issues or genetics, compared to cases where judges perceived the criminal behaviour of an offender to be obviously under his control (Carroll and Payne, 1977; Aspinwall *et al*., 2012).

What little literature that exists on judicial perceptions or decision making concerning ASDs specifically has not discussed how judges perceive diagnosed individuals, but has reported that recent court decisions have shown that judges have very limited knowledge and familiarity with hfASDs and ASDs (Freckelton and List, 2009). Recent court decisions have also demonstrated that judicial officers are most often not aware of the relevant characteristics of these disorders that are needed to make fair decisions on procedural issues, including criminal responsibility, concerning offenders with ASDs (Freckelton, 2013), and that judges require expert evidence and assistance to help them not only understand the disorders’ symptoms and how to factor them into decisions, but also how not to abuse or misuse the information presented (Freckelton and List, 2009). These conclusions are paired with past academic concerns that have stressed the need for better training on ASDs for criminal justice actors, including judges (Browning and Caulfield, 2011), as well as the presentation of effective and unbiased testimony from experts on ASDs in criminal trials (Freckelton and List, 2009).

Thus, to inform these issues, I present interview data that explore how judges perceive and understand hfASDs and their effects on an individual’s ability to formulate criminal intent and control his criminal actions. Interviews also probed judicial perceptions of how the presumed genetic origins of the disorders might constrain an individual’s ability to choose his thoughts, intentions, and actions. Here, I examine several emergent themes concerning hfASD offenders’ abilities to choose and regulate their behaviour, focusing on judicial perceptions of diagnosed offenders’ predispositions to criminal behaviour, their abilities to understand their surroundings and form criminal intent, and their lack of impulse control and difficulty controlling behaviour with regards to the potential genetic origins of hfASDs. As judges have an immense amount of power over the freedom, future, and treatment of offenders that pass through their courtrooms (Stewart, 2003), these findings aim to provide a window into how judges understand and perceive hfASDs as they relate to criminal behaviour, intent and liability, and correspondingly, how these perceptions may affect or be integrated into the opinions and decisions they make surrounding the culpability and sentencing of diagnosed offenders.

## Methods

The data reported here were collected and analysed according to the previously described method (Berryessa, 2014) during 21 semi-structured telephone interviews with California Superior Court Judges. The interviewed judges volunteered their participation after completing an anonymous survey on hfASDs and criminal offending. On the survey, participants had been given general information on hfASDs and their symptomatic features. Therefore, in addition to any past case or personal experiences with these disorders, our interview population had been briefed on these disorders before completing the interviews. The interviews were not contingent upon any questions or answers in the survey, and were deidentified as to be kept anonymous.

The interview guide used in this research included 20 questions in three categories:

1. genetic disorders, both generally and related to criminal offending;
2. ASDs and hfASDs, both generally and related to criminal offending; and
3. personal experiences with and media portrayal of hfASDs, both generally and in a criminal justice context.

The questions relevant to this paper were found in part two of the interview guide, including questions such as “What is your reaction when you hear that an offender has an hfASD?” and “What specific concerns do you have about hfASDs being used or seen in the criminal justice system?”.

The Stanford University Institutional Review Board approved this research and verbal consent was obtained from all interview subjects. Interviews were on average 25 minutes each, audio-recorded, transcribed, and entered into NVivo (Version 10) for qualitative coding. A coding scheme was established and validated (see Berryessa, 2014; Cohen *κ* = 0.89), and an inductive constant comparative approach was used for analysis (see Maykut and Morehouse, 1994).

This analysis focused on responses pertaining to the overarching coding category classified as “choice”. This category of codes captured how judges spoke in response to the semi-structured interview questions about how an offender’s hfASD or the genetics of the disorders might affect the ability of the individual to conscientiously direct actions, thoughts, or words. “Choice” responses were then coded into the following five sub-codes: “free will or determinism”, “genetic make-up or predisposition”, “impulse control or difficulty controlling behaviour”, “intention”, and “view of the world”, which were used to develop the three themes presented below. There were also several limitations to the research included in this analysis and its methods, which have been previously noted in comprehensive detail (Berryessa, 2014).

## Results

### Participants

Totally, 21 California Superior Court Judges ultimately participated in telephone interviews. In the USA, California Superior Courts are trial courts with jurisdiction over all criminal, including felonies and misdemeanors, and civil, including, family law, probate and juvenile, cases in the state of California. In some counties, special court departments handle mental health, drugs, small claims, and traffic cases. In total, there are 58 Superior Courts and 1,705 Superior Court Judges in the state of California. In 2011-2012, 8.5 million criminal and civil cases were filed in California Superior Courts[2].

Demographic and professional characteristics of interviewed judges are summarized in Table I. Our interview sample was representative of the gender and educational demographics of the entire California Superior Court Judge population (see footnote 2), but other demographics, such as age, time on bench, or cases handled per day, are not publically available for the total population.

### Interview findings

All 21 interviewed judges cited they had previous exposure, whether personal or professional, to hfASDs. Seven judges reported previous case experience with individuals with hfASDs, four citing they remembered multiple cases (interviews 5, 7, 10, 15) and three recalling just one case in their past professional experience (interviews 11, 13, 16). In total, 18 judges reported having a personal experience with individuals with hfASDs, ranging from family members to friends (interviews 1-10, 12, 15-21). Overall, interviewed judges’ views on the ways hfASDs influence an individual’s ability to make choices bearing on his criminal intent or behaviour fell into three main themes:

1. predisposition to behaviour;
2. the offender’s view of the world and criminal intention; and
3. offender’s difficulty controlling behaviour and lack of impulse control.

#### Predisposition to behaviour

In total, 13 judges’ had responses that focused on the prominent theme of predisposition to behaviour (interviews 1, 2, 5-8, 10, 11, 13, 15, 17, 19, 20), suggesting offenders suffering from hfASDs are predisposed to act in specific ways because of their disorders. This included six of seven judges with previous case experience involving hfASD offenders. These responses focused primarily on the genetic origins of the disorders and the offenders’ inabilities to “choose” to suffer from the disorders. One judge commented that “a part of being on this Autistic Spectrum is a genetic reason […] not something that is a choice” (interview 1), while another said, “This is a condition that clearly the defendant didn’t choose, it chose him” (interview 6). Additionally, one judge with multiple case experiences involving offenders with hfASDs highlighted that a diagnosed offender does not “even have a chance of coming up with some other outcome because of his disability or his predisposition” (interview 10).

Several judges expressed concern that this “predisposition” or lack of “choice” to suffer from the disorders noticeably affected their perceptions of these offenders and their behaviour, leading them to question how best to process and handle hfASD offenders in the criminal justice system. One response from a judge with hfASD case experience focused on added compassion after learning of an offender’s diagnosis with a hfASD: “I believe a lot of your destiny is determined by […] your cultural surroundings and your economic surroundings, but also your DNA, and I’m very, very sympathetic to people […] that are born with a certain mental health disorder, there’s nothing they can do about it so I’m very sympathetic” (interview 5).

The majority of judges’ reactions focused on their uncertainty regarding how to understand and make decisions regarding the criminal responsibility and sentencing of hfASD offenders: “This is the genetic makeup of this person. There is some question as to the degree of criminal responsibility that one may have with a certain genetic predisposition” (interview 1). Another judge who had seen multiple cases involving diagnosed offenders voiced hesitation on whether or not an offender’s criminal responsibility is actually affected by hfASDs: “I think that a lot of his behaviour was influenced by genes. You know, it creates a predisposition or a certain ease of neural pathways that lead to certain behaviours, but […] lack of responsibility is not the same as being caused by genetic makeup” (interview 15).

Regarding the sentencing of an offender with an hfASD diagnosis, one respondent with hfASD case experience stated, “I think it’s [hfASD disorder] an important factor. I mean if somebody is […] in front of me and we’re trying to figure out how they should be punished for something that they did, the question […] has to start with why did they do it? If they’re predisposed to doing it then, you know, that’s a problem” (interview 10). In the same vein, another judge who had been involved in one case with a diagnosed offender conveyed the idea of balancing the free will of an hfASD offender to act and proper criminal justice response: “I’m sure you’re familiar with the nature versus nurture […] and then you have the philosophical question of free will and so when you’re looking at how to sentence someone as well as criminal responsibility, you’re trying to balance obviously community safety with what causes are there beyond the person’s control” (interview 13).

#### The offender’s “view of the world” and criminal intention

Another major theme in eight of these interviews concerned how offenders “view the world” because of their diagnoses, and correspondingly, how their criminal intent is affected by those views and their disorders (interviews 3, 4, 9, 12, 13, 17, 19, 20). Many judges articulated that offenders with hfASDs inherently view the world differently because of their diagnoses, and that this affects how they perceive the actions and behaviour of those offenders. Judges, including those who had seen diagnosed offenders in their courtrooms, also described their understandings of how individuals with hfASDs recognize and react to the world: “I thought they didn’t really understand right and wrong from a moral standpoint, they don’t have typical human feelings” (interview 13). Another said, “They’re very bright, but […] they’re going to be a more isolated type of person, they’re not going to be social, they may be impacted by loud noises, or when confronted […] they may not be good at defending themselves verbally and that sort of thing […] they may shut down” (interview 17).

Several responses distinguished between how offenders with hfASDs process information and perceive the world vs “normal” criminal offenders. One judge commented that an hfASD diagnosis shows that an offender has difficulty “processing the information the same way another person would in terms of statements made by other people and their conduct” (interview 19). In the same vein, another judge said, “It’s the way they [hfASD offenders] perceive the world”, adding that the offender “will always be a person who just cannot understand the world the way everyone else does […] You can’t tell him he’s wrong because that is the way they think” (interview 3). The same judge also stressed that “it’s very important for me to know that his [an offender’s] interpretation of the world is different and that is the way he thinks”, because if he is “actually interpreting the entire situation wrong because of a mental disorder […] that is not going to want me […] to send him to prison […] what he did was he saw it differently than the rest of us because of something that’s wrong with him” (interview 3).

Judicial perceptions concerning how diagnosed offenders inherently view the world differently due to their disorders corresponded to judges’ responses regarding these offenders’ abilities to form criminal intent for their actions. Several judges commented that, since offenders with hfASDs perceive the world differently than “normal” offenders due to their diagnoses, it is difficult for judges to completely understand the role of intent in these offenders’ criminal actions as well as how to factor it into their rulings. One judge commented that an offender’s hfASD diagnosis creates “a significant impact in that so much of what I deal with, especially in the criminal realm. has to do with willful conduct […]. It’s the difference between […] being a crime and not being a crime, because if in the ‘willful malicious conduct’, [there is] no intent, then […] it’s not a crime” (interview 12).

Judges also described an hfASD diagnosis as a potential mitigating factor by bringing into question the presence of intent and a willful criminal act. One characteristic response was, “So the difference between there being a mitigating factor in what someone did versus their being willful and wanton act, without regard for […] the law, so I think it would have significant impact in my mind as how I would handle a case involving [a] high functioning autistic defendant” (interview 12).

#### Offender’s difficulty controlling behaviour and lack of impulse control

The final theme in nine of these interviews concerned hfASD offenders’ lack of impulse control and difficulty controlling their behaviour (interviews 2, 5, 6, 8, 11, 15, 16, 18, 19), including four responses from judges with previous hfASD case experience. One judge commented that an offender might have “no impulse control as a result of […] Asperger’s Syndrome […] It’s the condition that causes the behaviour or the lack of impulse control […] how does the fact that he has this disorder affect his ability to deal with stress […]. React to a certain situation. React to people that are […] perceived to be in his face. Control his impulses. Behave, you know, appropriately” (interview 2). Another who had seen multiple offenders with hfASDs in his courtroom said that the characteristics of these disorders can “make it difficult for him [an offender] to avoid being triggered into behaviour that would be very difficult for him to control” (interview 15). Unsurprisingly, as the responses within this theme focused on an offender’s ability or problems in controlling his behaviour due to his condition, seven of the nine judges represented in this current theme also were represented in responses’ discussing how offenders with hfASDs are predisposed to act in specific ways because of their conditions.

Many responses in this theme focused on how these elements might impact the evaluation of these offenders and responsibility determination for their actions in the courtroom: “I’d have to understand the nature of the […] disorder, how that impacts them as far as their ability to control their conduct and so forth […] As far as the criminal side of it, I’d want to know what the impact on criminal conduct was” (interview 8). Regarding culpability, one judge who had previously handled a case involving a diagnosed offender expressed that he perceives an offender suffering from an hfASD as “less culpable as somebody who has been given better mental health and better impulse control, better judgment. We’re always trained, it’s called the *mens rea*, did he intend to do evil. And when you can’t control your behaviour or you have difficulty controlling your behaviour based on your genetics, you know, you’re much more […] I’m much more sympathetic to somebody like that” (interview 5). Further, some responses focused on evaluating these offenders’ impulse control differently than that of other offenders: “I’ve had defendants with Asperger’s Syndrome and they’ve had anger impulses, they’ve had violent impulses, that I just evaluate differently than I do a person without the same condition” (interview 19).

One other area on which judges both with and without hfASD case experience often commented was how this lack of impulse control might cause danger to people that hfASD offenders come in contact with in the future, with responses like hfASDs “may make you more prone to violent behaviour if it’s not controlled […] if it is left unchecked, then you know, it creates a real dangerousness that I would be concerned about” (interview 11). Other judges had mixed feelings about how this potential danger affects their perceptions and feelings about these offenders (“It makes me very sympathetic towards them. On the other hand, I have to watch […] have to be careful […] it hurts them because they could be more dangerous than somebody who […] has more impulse control” (interview 5)), as well as the responsibility of these offenders (“It’s a two-edged sword because they’re less culpable because they’re not capable of controlling their impulses, but on the other hand, they can be more dangerous because of their impulse control … they’re more dangerous because they may do this in the future” (interview 5)).

## Discussion/conclusions

As a backdrop to this analysis, it is important to remember not every criminal act committed by offenders with hfASDs is due to or exculpable because of the characteristics of their disorders. Some criminal acts committed by those with hfASDs are still intentional criminal acts voluntarily perpetrated by the offenders and not due to the offenders’ lack of impulse control, their perceptions of the world, or their inability to control their behaviour. Every case involving an offender with an hfASD should be handled and assessed individually by the justice system and the assigned judge on a case-by-case basis, taking into account the characteristics and crimes of the individual defendant and how and if his disorder is relevant to his mental capacity, culpability, and the facts of the specific case at hand during the criminal process.

Even so, although each diagnosed offender and his actions should be assessed individually, the majority of individuals with hfASDs that exhibit criminal behaviour are thought to do so because of the characteristics of their disorders (Murrie *et al*., 2002; Barry-Walsh and Mullen, 2004; Howlin, 2004; Haskins and Silva, 2006; Attwood, 2006; Kristiansson and Sorman, 2008; Browning and Caulfield, 2011), which presents a complex set of issues for judges when handling diagnosed offenders. Judges interviewed in this analysis expressed a range of opinions regarding how offenders may be involuntarily predisposed to act in specific ways because of their hfASDs and the disorders’ potential genetic origins, including diagnosed offenders’ problems with impulse control and the abilities of these offenders to control their behaviour. These judges found these concepts to be challenging but significant to their future rulings and evaluations of offenders. Due to their perceptions, judges expressed hesitations on how to properly criminally process these offenders and make decisions regarding responsibility determination and sentencing.

Many judges also perceived that, due to their diagnoses, hfASD offenders inherently view the world and process information differently than other offenders, resulting in questions on how and if these offenders can properly formulate criminal intent for their actions. Judges found it difficult to completely understand the roles of and abilities of these offenders to formulate intent in many of these offenders’ criminal actions and how to consider it in their rulings, which can lead to questions whether hfASD offenders are exculpable from their actions because they are unable to exhibit the essential elements of a crime (*actus reus*, meaning “guilty act”, and *mens rea*, meaning “guilty mind”) necessary for liability. Although the opinions of judges with past hfASD experience were present within each interview theme, the attitudes expressed across our sample were relatively consistent in most areas regardless of judicial background, showing that judicial perceptions of hfASDs and corresponding questions or hesitations on how to process diagnosed offenders are true for both judges with and without past hfASD case experience.

Overall, these findings showed that regardless of past case experience, judges perceived that hfASD offenders “view the world” in a unique way, much of their behaviour is not directly under their control, and they are inherently different than other offenders and should be treated as such. Due to these attitudes, many judges cited the diagnosis as a potential mitigating factor in their perceptions and evaluation of intent, responsibility, and sentencing of diagnosed offenders, supporting past findings on the effects of offenders’ mental health on judicial attitudes and rulings (Hochstedler, 1987; Carroll and Payne, 1977). Yet, many judges also admitted to not knowing how to properly use information on these disorders in their decision making. As previous literature has reported (Freckelton and List, 2009; Freckelton, 2013), judges are limited in their ability to effectively and properly understand the disorders, their symptoms, and how to factor this information into their decisions in order to ensure they are fair and just.

The research included in this analysis study did have its limitations, as previously stated. The sample size was small and therefore the results were not statistically significant. In order to ensure anonymity, I was not able to collect or report some demographical data that might have helped to contextualize results but also potentially could have revealed judges’ identities, such as county of service or what types of cases, criminal or civil, these judges handle. All judges in the interview sample had been exposed either personally or professionally to hfASDs in the past; as preconceived perceptions and past experiences have been shown to affect attitudes and decisions of judges concerning mentally disordered offenders (Perlin and Gould, 1995; Wrightsman, 1999; Parry, 2005; Jones and Cauffman, 2008), opinions of interviewed judges may differ from those of the larger population. Additionally, besides gender and education, the other participant demographics collected in this study are unavailable for the entire population of California Superior Court Judges, and therefore, it is unknown how representative our sample is of the total population regarding those demographics.

Nevertheless, these findings provide insight on judicial understandings of hfASDs, the types of issues they identify as potentially challenging or influential when processing and making decisions concerning diagnosed offenders, areas of practice that could be affected, and a starting point for future research. This research should serve as fundamental insight for expert witnesses and those who run judicial training or educational programmes on ASDs or other mental health issues into some of the areas and types of difficulties judges have assessing information on hfASDs. These findings may aid experts better prepare testimony for cases involving diagnosed offenders to ensure judges are properly equipped to understand and handle these issues, as well as help mental health, justice, or other practitioners who run educational programmes for judges identify powerful or pertinent information and strategies that should be included in curricula or training on these issues. Even with past exposure to these disorders, many judges in our sample admitted to not being familiar or knowledgeable enough to effectively use information on hfASDs in their decision making. It seems judges do rely on expert assistance, whether it be in the courtroom or through a training programme, on these issues in order to make just decisions and not misuse information on these disorders (Freckelton and List, 2009; Freckelton, 2013), and therefore, these findings could be useful in those arenas.

Correspondingly, I recommend future research on expert witnesses in trials regarding hfASDs and types of evidence conveyed to judges. Future research should focus on experts who give testimony on these issues in trials, their professional backgrounds, types of information they choose to present, and what messages or concepts they aim to convey to the judiciary and the court. Also, future studies might wish to address the extent to which the effectiveness, quality, or types of experts or presented information on ASDs in trials involving diagnosed offenders affect case outcomes. Additionally, aligned with past recommendations (Browning and Caulfield, 2011), this research supports the need for and creation of meaningful training programmes on these issues for judges, as well as the criminal justice system as a whole, so judges are able to better understand these issues on their own and meet the needs of these individuals who are increasingly seen in the courtroom (Freckelton, 2013).

Finally, judges within this sample also discussed an hfASD diagnosis as a potential mitigating factor in their judgments regarding responsibility determination and sentencing, supporting suggestions that mental health factors mitigate judicial perceptions and decision making (Carroll and Payne, 1977; Hochstedler, 1987; Aspinwall *et al*., 2012). As this data only briefly and informally touched upon these concepts, I believe there are several lines of potential empirical research that could be undertaken to tangibly measure the ways in which these disorders mitigate judicial rulings on responsibility and sentencing. Future investigators may want to utilize case studies to survey judges and their rulings on sentence length or type for hfASD offenders compared to “normal” offenders concerning the same crime, or assess the real-life case outcomes of judges who have past hfASD case experience in order to properly inform these subjects.

## Figures and Tables

**Table I:** Judge demographic characteristics

Characteristic	Number and percentage of participants (n = 21)
*Age*	
< 30 years	0 (0.00%)
30-39 years	0 (0.00%)
40-49 years	1 (4.76%)
50-59 years	11 (52.38%)
60 + years	9 (42.86%)
*Gender*	
Female	7 (33.33%)
Male	14 (66.67%)
*Degree*	
MA	0 (0.00%)
JD	21 (100%)
PhD	0 (0.00%)
LLM	0 (0.00%)
MD	0 (0.00%)
MBA	1 (4.76%)
*Time on bench*	
< 1 year	2 (9.52%)
1-5 years	4 (19.05%)
6-10 years	7 (33.33%)
> 10 years	8 (38.10%)
*Cases handled per day*	
0-5 cases	5 (23.81%)
6-10 cases	2 (9.52%)
11-15 cases	1 (4.76%)
15 + cases	13 (61.91%)
